# Incidence and short-term outcomes of central line-related bloodstream infection in patients admitted to the emergency department: a single-center retrospective study

**DOI:** 10.1038/s41598-023-31100-1

**Published:** 2023-03-08

**Authors:** Hyun Min Ahn, June-sung Kim, Min Gul Park, Jeongeun Hwang, Won Young Kim, Dong-Woo Seo

**Affiliations:** 1 Department of Emergency Medicine, University of Ulsan College of Medicine, Asan Medical Center, 88, Olympic-ro 43-gil, Songpa-gu, Seoul, 05505 Korea; 2 Division of Medical Oncology, Department of Internal Medicine, Korea University College of Medicine, Seoul, Korea; 3 Department of Biomedical Research Center, Korea University Guro Hospital, Seoul, Korea; 4 Department of Information Medicine, University of Ulsan College of Medicine, Asan Medical Center, Seoul, Korea

**Keywords:** Infectious diseases, Medical research

## Abstract

Central line-related bloodstream infection (CRBSI) is a common complication during hospital admissions; however, there is insufficient data regarding CRBSI in the emergency department. Therefore, we evaluated the incidence and clinical impact of CRBSI using a single-center retrospective study to analyze medical data of 2189 adult patients (median age: 65 years, 58.8% males) who underwent central line insertion in ED from 2013 to 2015. CRBSI was defined if the same pathogens were identified at peripheral and catheter tips or the differential time to positivity was > 2 h. CRBSI-related in-hospital mortality and risk factors were evaluated. CRBSI occurred in 80 patients (3.7%), of which 51 survived and 29 died; those with CRBSI had higher incidence of subclavian vein insertion and retry rates. *Staphylococcus epidermidis* was the most common pathogen, followed by *Staphylococcus aureus*, *Enterococcus faecium*, and *Escherichia coli*. Using multivariate analysis, we found that CRBSI development was an independent risk factor for in-hospital mortality (adjusted odds ratio: 1.93, 95% confidence intervals: 1.19–3.14, *p* < 0.01). Our findings suggest that CRBSI after central line insertion in the emergency department is common and associated with poor outcomes. Infection prevention and management measures to reduce CRBSI incidence are essential to improve clinical outcomes.

## Introduction

Central venous catheterization is a frequently employed technique for monitoring and managing critically ill patients^1^. Owing to the recent surge in the frequency of hospital visits and admissions through the emergency room for critically ill patients, central line insertion is commonly used for continuous infusion of ionotropic agents, repeated blood samplings, and real-time monitoring^2^. Consequently, there is also an increased incidence of central line insertion-related side effects, including mechanical problems (such as pneumothorax, remnant guidewire, and external puncture) and infectious problems, which negatively affect the patient's clinical outcomes^3,4^. One such complication, central line-related bloodstream infection (CRBSI), is an important cause of nosocomial infection^4^.

Previous epidemiologic studies have reported that CRBSIs are still fairly common and associated with poor prognosis for critically ill patients admitted to the intensive care unit (ICU)^5^. A study based on the patients admitted general ward reported that infection with *Staphylococcus aureus* or *Candida* and a Charlson Comorbidity score > 4 were independent risk factors for 30-day mortality^6^. Furthermore, critically ill patients, who have to frequently visit and stay in the emergency department (ED), may be more susceptible to CRBSI because of their unique scenarios and associated difficulty in maintaining a sterile atmosphere during frequent ED visits. Although there are several reports about the incidence of CRBSI in ED, these studies lack statistical power because of the small sample sizes^7^.

Therefore, in this study, we performed single center, retrospective, cohort study to evaluate the incidence of CRBSI among patients who receive a central line insertion in ED to identify clinical factors associated with CRBSI and assess its impact on in-hospital mortality.

## Results

**Figure 1:**
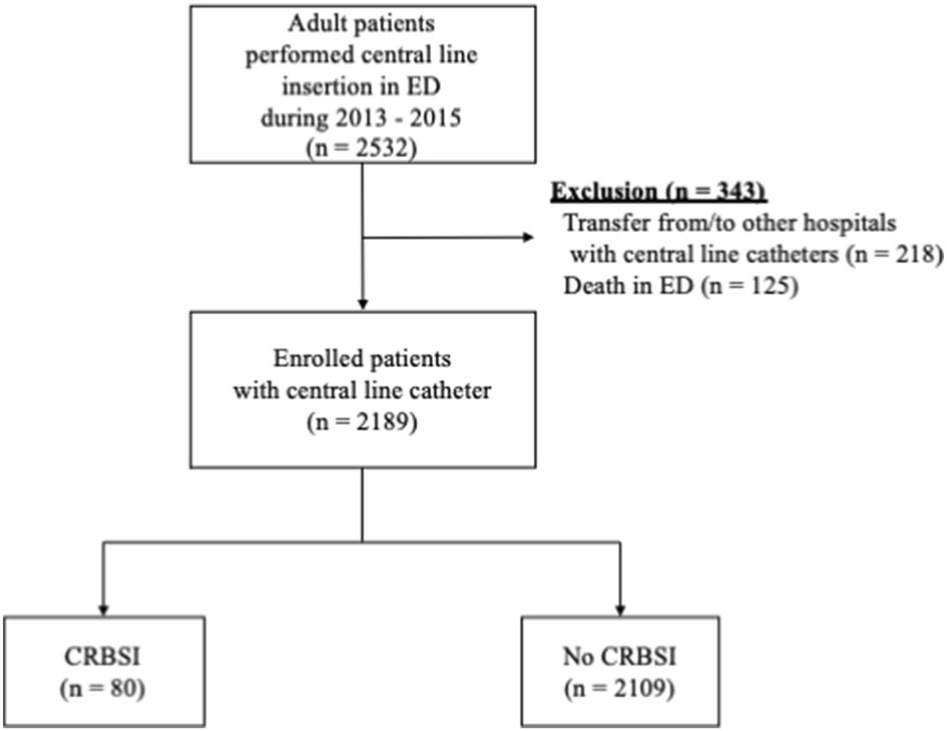
Flowchart of the study sample recruitment process.

From March 2013 to February 2015, 2532 patients were found eligible for the study. After excluding 343 patients, who were either transferred from/to other hospitals with central line insertion or died in ER, we analyzed data for 2189 patients (median age: 65 years; 58.8% males) (Fig. 1). Table 1 summarizes the demographic data, medical history, and central line insertion-related information for these patients. Out of these 2189 patients, 80 (3.7%) experienced CRBSI during their hospital stay.

**Table 1:** Baseline characteristics of the study population.

Characteristics	Total (n = 2189)	No CRBSI (n = 2109)	CRBSI (n = 80)	*P* value
Age	65.0 (54.0–74.0)	66.0 (56.0–74.0)	68.0 (56.0–75.0)	< 0.01
Male	1288 (58.8)	1239 (58.7)	49 (61.3)	0.66
TPN	567 (25.9)	527 (25.0)	40 (50.0)	< 0.01
Hematologic malignancy*	96 (4.4)	90 (4.3)	6 (7.5)	0.17
Transplantation**	86 (3.9)	80 (3.8)	6 (7.5)	0.09
Hemodialysis	234 (10.7)	211 (10.0)	23 (28.8)	< 0.01
Catheter-related information				
Right-side insertion	1759 (80.4)	1697 (80.5)	62 (77.5)	0.51
Internal jugular vein	1762 (80.5)	1699 (80.6)	63 (78.8)	0.69
Subclavian vein	273 (12.5)	265 (12.6)	8 (10.0)	0.50
Femoral vein	142 (6.5)	133 (6.3)	9 (11.3)	0.08
Retry***	62 (2.8)	56 (2.7)	6 (7.5)	0.01
In-hospital death	467 (21.3)	438 (20.8)	29 (36.3)	< 0.01

Data are presented as the median (interquartile range) or the frequency (percentage).

*Hematologic malignancy included leukemia, lymphoma, myelodysplastic syndrome, and multiple myeloma.

**Transplantation included heart, lung, liver, pancreas, and kidney.

***Retry was defined as any insertion trial beyond the first two trials.

*CRBSI* central line-related blood stream infection, *TPN* total parenteral nutrition.

In comparing patient groups with and without CRSBI, we found that the former had a higher proportion of patients receiving total parenteral nutrition (TPN) during hospital admission than those without CRBSI (50% versus 25%). Also, the CRBSI group had a greater incidence of transplantations (7.5% versus 3.8%) and hemodialysis (12.5% versus 4.9%). Most of the patients underwent right-sided insertion (80.4%) within the internal jugular vein (80.5%). Femoral vein approach and retry rates were more common in patients with CRBSI than without CRBSI (11.3% versus 6.3%, and 7.5% versus 2.7%, respectively). A total of 165 pathogens were identified from the 80 patients diagnosed with CRBSI (Table 2). The most common microorganism was *Staphylococcus epidermidis* (n = 22, 13.3%), followed by *Staphylococcus aureus* (n = 17, 10.3%), *Enterococcus faecium* (n = 14, 8.5%), and *Escherichia coli* (n = 13, 7.9%).

**Table 2:** Frequency distribution of different bacteria in culture-positive results as the cause of infection.

Infections species	Frequency (%)
*Staphylococcus epidermiditis*	22 (13.3)
*Staphylococcus aureus*	17 (10.3)
*Enterococcus faecium*	14 (8.5)
*Escherichia coli*	13 (7.9)
*Candida albicans*	13 (7.9)
*Pseudomonas aeruginosa*	11 (6.7)
*Klebsiella pneumoniae*	8 (4.8)
*Acinetobacter baumanni*	8 (4.8)
*Candida glabrata*	8 (4.8)
*Enterococcus faecalis*	5 (3.0)
Others	17 (10.3)

Data are presented as n (%).

**Figure 2:**
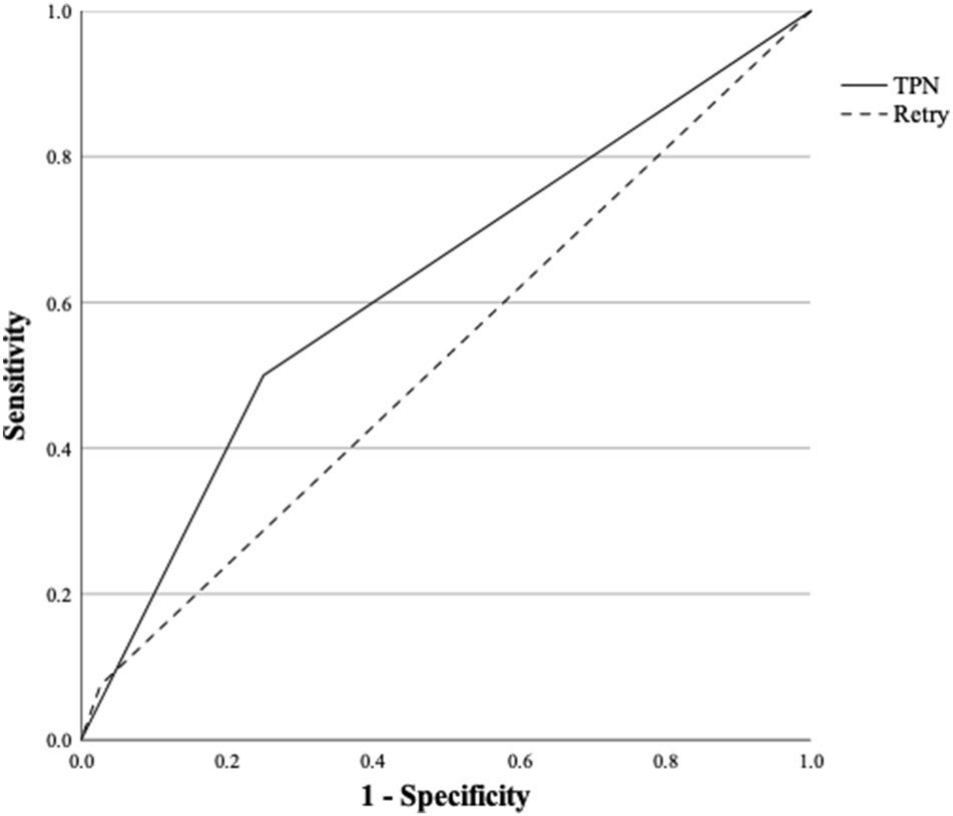
Receiver operand characteristic (ROC) curve for the occurrence of central-line-related bloodstream infection (CRBSI).

In the multivariate logistic regression model, TPN and retry rates were found to be independent risk factors for developing CRBSI during hospital admission (Table S1). Figure 2 shows a receiver operand characteristic (ROC) curve for TPN and retry rates regarding the development of CRBSI. The area under the curve (AUC) for TPN and retry rates were 0.625 and 0.524, respectively.

There was a 21.3% (n = 467) in-hospital mortality rate in our study sample. The baseline characteristics of the study population stratified according to in-hospital deaths are presented in Table S2. Table 3 presents the results of univariate and multivariate analyses of in-hospital mortality of patients with CRBSI. As per the univariate analysis, we found that the following factors were statistically different between survivors and non-survivors—age, male sex, hematologic malignancy, hemodialysis, incidence of retrials, right-sided insertion, internal jugular vein insertion, subclavian vein insertion, and CRBSI. According to the multivariate analysis, the development of CRBSI was an independent risk factor for in-hospital death (adjusted odds ratio, OR 1.93, 95% confidence intervals, CI 1.19–3.14, *p* < 0.01).

**Table 3:** Univariate and multivariate logistic regression model for predicting in-hospital mortality.

Variables	Univariable			Multivariable		
	OR	95% CI	*P*	Adjusted OR	95% CI	*P*
Age	1.02	1.01–1.02	< 0.01	1.02	1.01–1.02	< 0.01
Male	1.17	0.74–1.45	0.16			
Hematologic malignancy	1.68	1.35–2.30	< 0.01	1.85	1.17–2.93	< 0.01
Hemodialysis	1.28	1.18–1.45	< 0.01	1.28	1.17–1.44	< 0.01
Retry	1.87	0.98–3.56	0.06	1.84	0.97–3.47	0.06
Right-side insertion	0.55	0.37–0.82	0.04	0.56	0.38–0.83	< 0.01
Internal jugular vein	0.69	0.18–2.59	0.58	0.55	0.34–0.89	0.02
Subclavian vein	0.64	0.17–2.33	0.50	0.52	0.29–0.95	0.03
Femoral vein	1.26	0.32–4.91	0.74			
CRBSI	1.95	1.20–3.18	< 0.01	1.93	1.19–3.14	< 0.01

*OR* odds ratio, *CI* confidence interval, *CRBSI* central line-related blood-stream infection.

## Discussion

In this retrospective cohort study, we found that only 3.7% of patients who received a central line catheter insertion in ED developed CRBSI, and the occurrence of CRBSI was an independent risk factor for in-hospital mortality. These findings suggest that CRBSI is a relatively common and serious complication in patients who have to undergo central line insertion in ED.

Despite the increasing awareness about CRBSI-associated mortality, morbidity, and excess medical burden, the incidence of this nosocomial infection is increasing, ranging from 3 to 20% depending on the study population^8–10^. A recent population-based study reported that CRBSI occurred in 19.2% of patients with suspected systemic inflammatory response syndrome^11^. However, there is only limited data exploring the incidence of CRBSI in patients who undergo catheter insertion in the ED. Although we could not collect detailed clinical data, such as the severity of the acute illness, specific diagnosis, and indications of central line catheterization, the incidence of CRBSI was quite similar to that reported by previous research^12,13^. This result might imply that the occurrence of CRBSI did not markedly increase in the ED environments and could be controlled if sterilization and management techniques were strictly applied. Additionally, the risk factors for occurring CRBSI, including age, TPN, hemodialysis, and repeated trials, have also been cited in the literature^14,15^. Notably, repeated trials for insertion were the most potent predictor for mortality (adjusted OR 3.11, 95% CI 1.28–7.53). This finding underscores the importance of efforts to place the catheter in a single trial, such as ultrasound-guided access, for reducing the incidence of CRBSI^16^.

Saliba et al.^6^ found that *Staphylococcus* species were the most common causative microorganism for CRBSI, which was also observed in our study—notably *Staphylococcus epidermidis* was more common than *Staphylococcus aureus*. Furthermore, in cases where longer maintenance of catheterization (usually > 10 days) is required, endoluminal spread from the catheter hub is a known pathway for infection^17^. Colonization by cutaneous pathogens along the external skin of the catheter was found to be the main cause of bacteremia in our study. Therefore, sterilization efforts for strengthening the infection bundle, including hand hygiene, aseptic skin preparation, and avoidance of inappropriate insertion sites, must be enforced to reduce CRBSI after catheterization in ED^18,19^.

Owing to the extensive research and implementation of aggressive monitoring and management strategies in patients with suspected CRBSI, CRBSI-associated mortality has decreased considerably over recent years^20,21^. Nevertheless, the correlation between the occurrence of CRBSI and greater mortality is inconsistent. Similar to previous studies, we also found that the development of CRBSI was an independent risk factor for in-hospital mortality^22,23^. On the other hand, other retrospective cohort studies revealed that CRBSI was not an attributable factor in increasing ICU mortality^11,24^. There are multiple plausible explanations for this. First, the characteristics of the study populations are quite different from each other since age, underlying illnesses, and severity of active disease could have affected the mortality. Second, the management of patients with suspected CRBSI, including the timing of catheter removal and selection of empirical antibiotics, was not identical. Accordingly, future research must utilize a randomized controlled study design to provide high-level evidence about the association between mortality and CRBSI.

There were some limitations to our study. This was a single-centered retrospective study design which limits the generalizability of our results. The severity of disease, the patient's clinical profile in the ED, and the expertise of professionals dispensing these interventions would differ in each hospital. Moreover, we could not exclude several hidden confounding factors, such as sterile technique during insertion and manage, and include information about additional treatment outside ED, including central line changes. Despite the limitations, the strength of our study is its relatively large sample size of patients and the inclusion of various factors in the multivariate analysis of CRBSI and in-hospital mortality.

In conclusion, we found that CRBSI after central line insertion in the ED is a fairly common complication and is associated with poor outcomes such as in-hospital mortality. Management and prevention measures to reduce the occurrence of CRBSI are warranted to improve both clinical and patient outcomes.

## Methods

### Study design and population

This retrospective cohort study was conducted at the ED of a tertiary care university-affiliated hospital in Seoul, Korea, which has an annual turnover of about 110,000 visits. The Asan Medical Center Institutional Review Board approved this study before its commencement (Approval No.: 2016-0081) and waived the requirement for informed consent due to the retrospective nature of the study. All methods were performed in accordance with the relevant guidelines and regulations. All adult patients (aged ≥ 18 years), who had a central venous catheter insertion in the ED from March 2013 to February 2015, were included in this study. Patients transferred to/from other hospitals with central venous catheters, and those who died in the ED or were discharged were excluded.

### Data collection

In the study facility, insertion of the central line catheterization was decided by ED physicians on duty. The technique of central venous catheterization was followed by the standard practice (i.e., Seldinger Technique). In brief, physicians were positioned a patient’s head for internal jugular vein and subclavian vein, and leg for femoral vein. After turning the head or leg away from the intended site of access, decontamination of the skin with an chlorhexidine-alcohol was performed. Ultrasound was used to evaluate the vein position, patency, and adjacent anatomy prior to access. Needle was punctured and guidewire was inserted followed by the dilator advancement. The actual catheter insertion was done over the guidewire and routine x-ray was conducted to find complications such as pneumothorax, malposition, and remnant guidewire.

Demographic and clinical data including age, sex, comorbidities, laboratory investigation results on ED admission, and clinical outcomes including in-hospital mortality were extracted from the hospital’s electronic medical records and the Asan BiomedicaL research Environment database, which is a system to anonymizes medical data used for research in the study facility. Moreover, information regarding central venous catheter insertion, such as site of insertion, and retry, were also extracted. Retry was defined as any insertion trial beyond the first two trials which was recorded routinely by nurses on duty.

For defining CRBSI, the following two conditions were to be satisfied: (1) same microbes discovered from peripheral venous culture and from quantitative (> 15 colony-forming units, CFU) culture of the central catheter tip; or (2) a shorter time to a positive result (> 2 h earlier) in the central venous catheter sample than the peripheral sample based on data of differential time to positivity^25–27^. In the event of co-infections, we counted each pathogen individually. We counted recurrent or repeated cases individually if patients were discharged and readmitted, as we hypothesized that CRBSI was completely resolved between the discharges and next admissions. In the case of repeated cultures during a single admission, we counted them as a single event and included in analyses. The primary outcome was the association between CRBSI and in-hospital mortality after admission to the ED; secondarily, we evaluated the risk factors for CRBSI.

### Statistical analysis

Since the data were non-normally distributed, continuous variables are presented as medians with inter-quartile ranges (IQRs); categorical variables are presented as frequency and percentages. The Mann–Whitney U test was used to compare continuous variables, and the chi-square or Fisher’s exact tests were used to compare categorical variables. ROC curves with AUC were calculated as conventional methods for predicting the incidence of CRBSI. For calculation of OR, we used backward stepwise multiple logistic regression analysis with statistically different variables (*p* < 0.1) in univariate analysis. A two-sided *p* value < 0.05 was considered statistically significant. All statistical analyses were performed using R (version 4.1.3; R Foundation for Statistical Computing, Vienna, Austria).

## Supplementary Information

Supplementary Tables.

## Data Availability

The datasets of this study can be obtained from the corresponding author on reasonable request.

## References

[1] Biffi R (2009). Best choice of central venous insertion site for the prevention of catheter-related complications in adult patients who need cancer therapy: A randomized trial. Ann. Oncol..

[2] Jamshidi R (2019). Central venous catheters: Indications, techniques, and complications. Semin. Pediatr. Surg..

[3] Ullman AJ, Marsh N, Mihala G, Cooke M, Rickard CM (2015). Complications of central venous access devices: A systematic review. Pediatrics.

[4] Gahlot R, Nigam C, Kumar V, Yadav G, Anupurba S (2014). Catheter-related bloodstream infections. Int. J. Crit. Illn. Inj. Sci..

[5] Wong SW (2016). The influence of intensive care unit-acquired central line-associated bloodstream infection on in-hospital mortality: A single-center risk-adjusted analysis. Am. J. Infect. Control.

[6] Saliba P (2018). Mortality risk factors among non-ICU patients with nosocomial vascular catheter-related bloodstream infections: A prospective cohort study. J. Hosp. Infect..

[7] Theodoro D (2015). Emergency department central line–associated bloodstream infections (CLABSI) incidence in the era of prevention practices. Acad. Emerg. Med..

[8] Badia-Cebada L (2022). Trends in the epidemiology of catheter-related bloodstream infections; Towards a paradigm shift, Spain, 2007 to 2019. Eurosurveillance.

[9] Rupp ME, Karnatak R (2018). Intravascular catheter–related bloodstream infections. Infect. Dis. Clin. N. Am..

[10] Mushtaq A (2018). Comparison of complications in midlines versus central venous catheters: Are midlines safer than central venous lines?. Am. J. Infect. Control.

[11] Zhong Y (2021). Incidence, risk Factors, and attributable mortality of catheter-related bloodstream infections in the intensive care unit after suspected catheters infection: A retrospective 10-year cohort study. Infect. Dis. Ther..

[12] Lendak D (2021). Changing epidemiology of catheter-related bloodstream infections in neutropenic oncohematological patients. PLoS ONE.

[13] Zingg W (2009). Hospital-wide surveillance of catheter-related bloodstream infection: From the expected to the unexpected. J. Hosp. Infect..

[14] Bell T, O’Grady NP (2017). Prevention of central line–associated bloodstream infections. Infect. Dis. Clin. N. Am..

[15] Merrill KC, Sumner S, Linford L, Taylor C, Macintosh C (2014). Impact of universal disinfectant cap implementation on central line–associated bloodstream infections. Am. J. Infect. Control.

[16] Schmidt GA (2019). Ultrasound-guided vascular access in critical illness. Intensive Care Med.

[17] Fätkenheuer G, Cornely O, Seifert H (2002). Clinical management of catheter-related infections. Clin. Microbiol. Infect..

[18] Lee KH (2018). Effect of central line bundle compliance on central line-associated bloodstream infections. Yonsei Med. J..

[19] Ullman AJ (2015). Dressings and securement devices for central venous catheters (CVC). Cochrane Database Syst. Rev..

[20] Chou EH (2020). Incidence, trends, and outcomes of infection sites among hospitalizations of sepsis: A nationwide study. PLoS ONE.

[21] Stevens V (2014). Inpatient costs, mortality and 30-day re-admission in patients with central-line-associated bloodstream infections. Clin. Microbiol. Infect..

[22] Magill SS (2014). Multistate point-prevalence survey of health care–associated infections. N. Engl. J. Med..

[23] Siempos II, Kopterides P, Tsangaris I, Dimopoulou I, Armaganidis AE (2009). Impact of catheter-related bloodstream infections on the mortality of critically ill patients; A meta-analysis. Crit. Care Med..

[24] van Vught LA (2016). Incidence, risk factors, and attributable mortality of secondary infections in the intensive care unit after admission for sepsis. JAMA.

[25] Mermel LA (2009). Clinical practice guidelines for the diagnosis and management of intravascular catheter-related infection: 2009 update by the Infectious Diseases Society of America. Clin. Infect. Dis..

[26] Maki DG, Weise CE, Sarafin HW (1977). A semiquantitative culture method for identifying intravenous-catheter-related infection. N. Engl. J. Med..

[27] Raad I (2004). Differential time to positivity: A useful method for diagnosing catheter-related bloodstream infections. Ann. Intern. Med..

