# Supplementary material for: Incidence and short-term outcomes of central line-related bloodstream infection in patients admitted to the emergency department: a single-center retrospective study
Source: Sci Rep. 2023 Mar 8;13:3867. doi: 10.1038/s41598-023-31100-1 (PMC9995448; doi:10.1038/s41598-023-31100-1)
Supplement: Supplementary file 1 — Supplementary Tables. [file 41598_2023_31100_MOESM1_ESM.pdf]

Table S1. Univariate and multivariate logistic regression model for predicting CRBSI

Variables	Univariable			Multivariable		
	OR	95% CI	*P*	Adjusted OR	95% CI	*P*
Age	1.01	0.97 – 1.03	0.14			
TPN	2.94	1.87 – 4.64	< 0.01	3.03	1.93 – 7.75	0.01
Hemodialysis	1.42	0.98 – 1.96	0.82			
Retry	2.88	1.17 – 7.09	0.02	3.11	1.28 – 7.53	0.01
Transplantation	2.29	0.93 – 5.65	0.07			

Abbreviations: OR = odds ratio; CI = confidence interval; TPN = total parenteral nutrition; CRBSI = central line-related blood stream infection.

Table S2. Baseline characteristics of the study population according to in-hospital deaths

Characteristics	Total (n = 2189)	Survival (n = 1722)	Non-survival (n = 467)	*P*-value
Age	65.0 (54.0 – 74.0)	65.0 (53.0 – 73.0)	67.0 (56.0 – 75.0)	< 0.01
Male	1288 (58.8)	996 (57.8)	292 (62.5)	0.07
TPN	567 (25.9)	450 (26.1)	117 (25.1)	0.64
Hematologic malignancy*	96 (4.4)	66 (3.8)	30 (6.4)	0.02
Transplantation**	86 (3.9)	74 (4.3)	12 (2.6)	0.09
Hemodialysis	289 (13.2)	157 (9.1)	132 (28.3)	< 0.01
Right side	1759 (80.4)	1419 (82.4)	340 (72.8)	< 0.01
Internal jugular vein	1762 (80.5)	1418 (82.3)	344 (73.7)	< 0.01
Subclavian vein	273 (12.5)	196 (11.4)	77 (16.5)	< 0.01
Femoral vein	142 (6.5)	100 (5.8)	42 (9.0)	0.01
Retry***	62 (2.8)	36 (2.1)	26 (2.6)	< 0.01
CRBSI	80 (3.7)	51 (3.0)	29 (6.2)	< 0.01

Data are presented as the median (interquartile range) or the frequency (percentage).

*Hematologic malignancy included leukemia, lymphoma, myelodysplastic syndrome, and multiple myeloma.

*Transplantation included heart, lung, liver, pancreas, and kidney.

***Retry was defined as any insertion trial beyond the first two trials.

Abbreviations: CRBSI = Central line-related blood stream infection; TPN = total parenteral nutrition.
